# Tumor marker pseudoprogression and immune-related cholangitis during conversion therapy for massive hepatocellular carcinoma: a case report

**DOI:** 10.3389/fimmu.2025.1529016

**Published:** 2025-06-04

**Authors:** Xiaodong Zhang, Luyi Zhang, Shuangying Zhao, Lingling Dai, Huayu Li, Xudong Wu, Huanjun Yan, Rongfeng Lin, Kelei Zhu

**Affiliations:** ^1^ Department of Hepatopancreatobiliary Surgery, The Affiliated People’s Hospital of Ningbo University, Ningbo, China; ^2^ Zhejiang Provincial Key Laboratory of Pathophysiology, Health Science Center, Ningbo University, Ningbo, China; ^3^ Department of Pathology, The Affiliated People’s Hospital of Ningbo University, Ningbo, China; ^4^ Department of Radiology, The Affiliated People’s Hospital of Ningbo University, Ningbo, China

**Keywords:** immune checkpoint inhibitor, tislelizumab, immune-related adverse event, tumor marker, pseudoprogression, hepatocellular carcinoma, conversion therapy, case report

## Abstract

Cases with massive (diameter ≥10 cm) hepatocellular carcinomas (HCCs) are uncommon and typically have poor outcomes; however, conversion therapy offers a beacon of hope for remission in patients with massive unresectable HCCs. Recently, immune checkpoint inhibitors (ICIs) have been used in combination with other treatment modalities to improve the response rates to conversion therapies, yet the safety and generalizability of this combination have not been extensively validated. Herein, we report a man with a chief complaint of abdominal pain who was diagnosed with massive unresectable HCC. Notably, the patient successfully underwent curative surgery after quadruple conversion therapy using tislelizumab (an ICI), lenvatinib, transarterial chemoembolization, and hepatic arterial infusion chemotherapy directed by a multidisciplinary team. With a complete response achieved, this case demonstrated the major potential of this combination regimen for HCC, and the remarkable efficacy was also reflected by substantial reductions in both alpha-fetoprotein and des-gamma-carboxy prothrombin overall. Nevertheless, transient increases in both biomarkers (tumor marker pseudoprogression) were observed within the first three weeks after initiating ICI treatment. Furthermore, the patient developed a biliary stricture, which resolved after discontinuing the ICI and was ultimately assessed as an immune-related adverse event. Therefore, in the context of combination therapy, further evaluation of the robustness of tumor markers is warranted, and it is crucial for clinicians to be mindful of potential immune-related adverse events.

## Introduction

Hepatocellular carcinoma (HCC) with a single lesion exceeding 10 cm, known as massive HCC, is not only uncommon and challenging to resect but also associated with a less favorable prognosis (1, 2). Conversion therapy, an emerging therapeutic strategy, may improve the prognosis of patients suffering from massive unresectable HCC (3). Recent years have witnessed the emergence of various conversion regimens for HCC, which typically involve tyrosine kinase inhibitors (TKIs) and locoregional therapies—transarterial chemoembolization (TACE) and hepatic arterial infusion chemotherapy (HAIC) (3–5). However, their efficacy and safety remain controversial, and there are currently no well-recognized preferred conversion regimens (3–5).

Immune checkpoint inhibitors (ICIs) have achieved encouraging results since they were approved for systemic therapy for HCC in 2020 (6, 7). Nonetheless, pseudoprogression, albeit infrequent, and immune-related adverse events (irAEs) raise new challenges for clinicians, yet no validated biomarkers are available to guide clinical decisions (6, 8). Furthermore, because of inadequate monitoring, the incidence of hepatobiliary irAEs is underestimated (9). More recently, better efficacy has been reported in combining ICIs with TACE, HAIC, and TKIs as quadruple conversion therapy compared with TACE alone, but the safety of this combination is largely unknown (10, 11). The case reported here underscores these knowledge gaps as our patient exhibited uncommon pseudoprogression of tumor markers and immune-related cholangiopathy during the quadruple conversion therapy.

## Case presentation

On 14 February 2023, a 43-year-old man presented to our outpatient department with right upper quadrant abdominal pain. He had been chronically infected with hepatitis B virus (HBV) for more than 20 years but had not taken entecavir regularly. His other medical history was unremarkable. Physical examination and ultrasound suggested a large space-occupying lesion in his right hemiliver; hence, he was admitted to the hospital.

**Figure 1:**
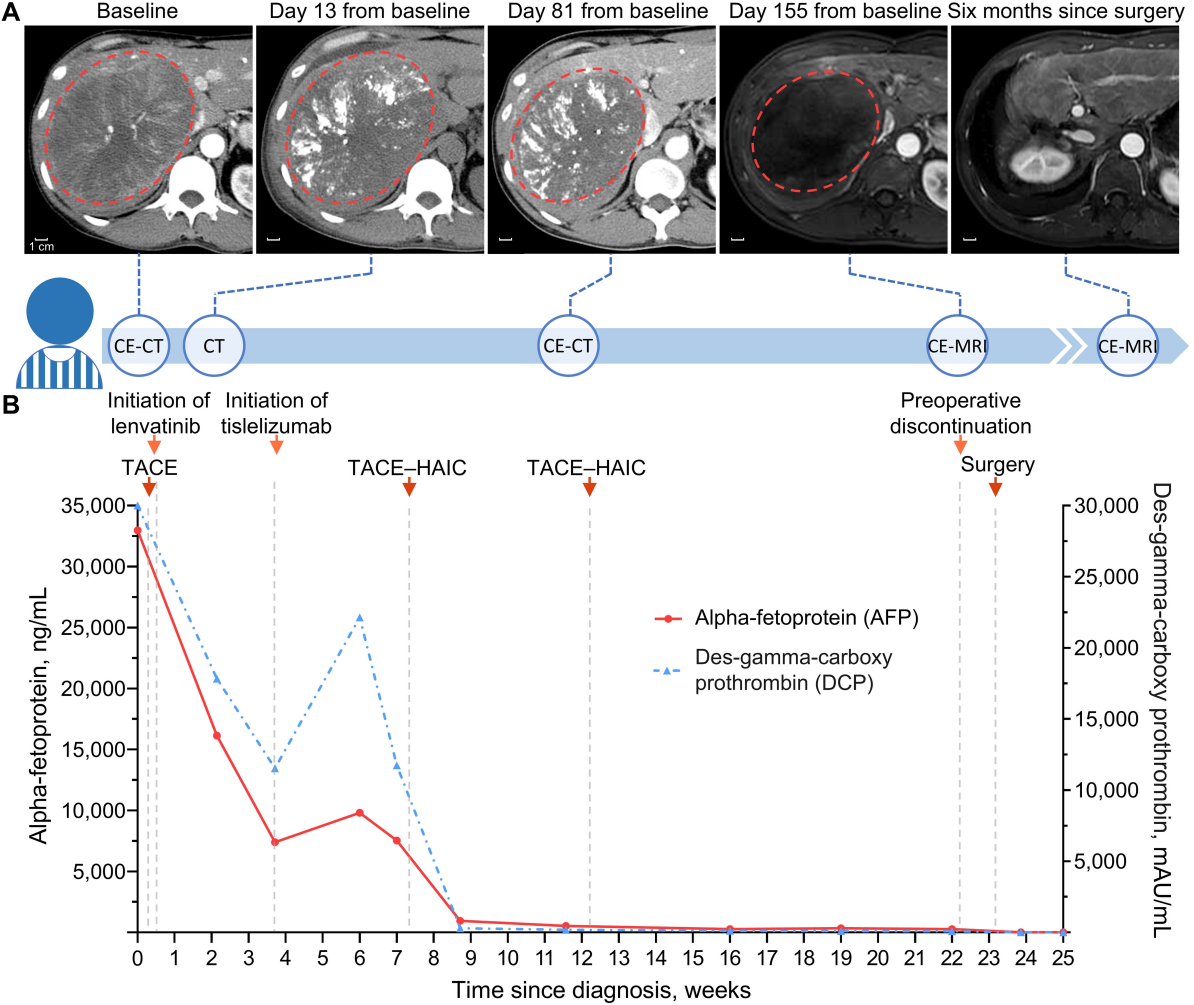
Timeline of clinical treatment and assessment. **(A)** Serial scans along the treatment timeline with red dotted circle depicting the dominant tumor lesion. Contrast-enhanced scans were obtained in the arterial phase, and no intratumoral arterial enhancement remained in the liver on day 155 from baseline. **(B)** Line graph showing changes in tumor markers during conversion therapy. Baseline DCP value above the upper limit of detection, 30,000 mAU/mL, is truncated to 30,000 mAU/mL. CE-CT, contrast-enhanced computed tomography; CE-MRI, contrast-enhanced magnetic resonance imaging; TACE, transarterial chemoembolization; HAIC, hepatic arterial infusion chemotherapy.

Laboratory workup revealed elevated HBV DNA—3.21×10^6^ IU/mL—and tumor markers, including alpha-fetoprotein (AFP) 32,982 ng/mL and des-gamma-carboxy prothrombin (DCP, also known as PIVKA-II) >30,000 mAU/mL. Subsequent contrast-enhanced computed tomography demonstrated a massive mass—13.8×11.0×13.6 cm—in the right lobe of his liver and several scattered small nodules throughout the liver (**Figure 1A**), whereas no abnormalities were found in other organs. Additionally, his Eastern Cooperative Oncology Group status was zero, and his Child–Pugh score was six. Taken together, he was diagnosed with intermediate-stage HCC according to the Barcelona Clinic Liver Cancer classification (12).

Due to the high HBV load, he started antiviral treatment immediately with 0.5 mg of oral entecavir once daily (13). He was then evaluated by a multidisciplinary team (MDT) and recommended the quadruple conversion regimen comprising TACE, TKI, ICI, and HAIC (14). Upon consent, he was treated with the conversion regimen, followed by serial imaging and biomarker measurements to track treatment response (**Figures 1A, B**). The following is a summary description of the treatment process. On 17 February, he underwent the first TACE, for which 5-fluorouracil (250 mg) and epothilone (50 mg) were mixed 1:2 with lipiodol. Lenvatinib (a TKI; 8 mg once daily) was taken orally beginning on 18 February, and tislelizumab (an ICI; 200 mg every 3 weeks) was administered intravenously beginning on 12 March. On 6 April and 10 May, he received two cycles of TACE–HAIC; for TACE, 5-fluorouracil (250 mg) and epothilone (30 mg) were mixed 1:2 with lipiodol; for HAIC, which was performed following TACE, raltitrexed (4 mg/m^2^ infusion for 1 h) and oxaliplatin (100 mg/m^2^ infusion for 4 h) were used.

**Figure 2:**
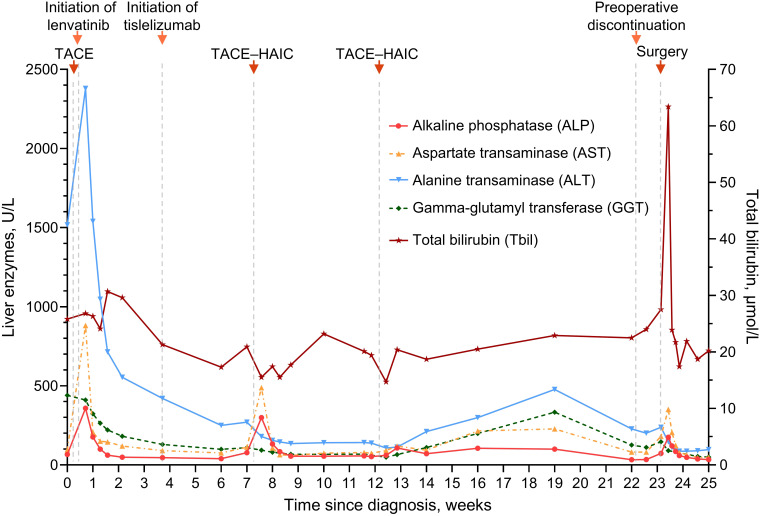
Line graph showing changes in liver enzymes and total bilirubin during conversion therapy. TACE, transarterial chemoembolization; HAIC, hepatic arterial infusion chemotherapy.

Remarkably, there were sharp increases followed by spontaneous decreases in both AFP and DCP within three weeks after the first dose of the ICI—tislelizumab (**Figure 1B**). It was ultimately assessed as tumor marker pseudoprogression since follow-up imaging did not reveal disease progression (15). Notwithstanding several adverse events such as aspartate aminotransferase and alkaline phosphatase increase (**Figure 2**), they were resolved with symptomatic treatment (13, 16). Overall, his vital signs were stable during the conversion therapy.

**Figure 3:**
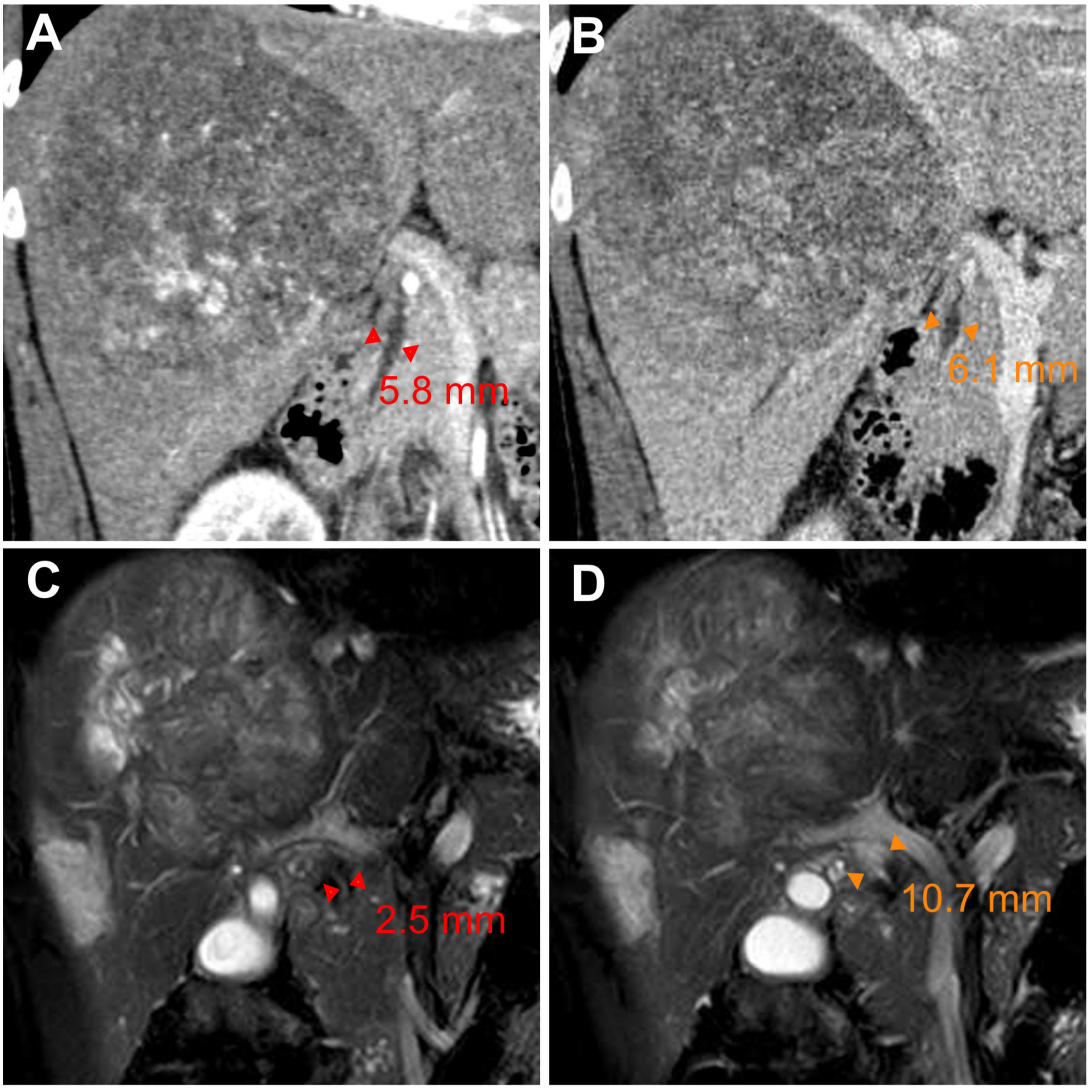
Radiologic scans of the common bile duct before and after conversion therapy. **(A, B)** Coronal multiplanar reconstruction of contrast-enhanced computed tomography images displaying the pretreatment (baseline) diameter of the middle-lower **(A)** and upper **(B)** portions of the common bile duct. **(C, D)** Magnetic resonance cholangiopancreatography images revealing **(C)** stricture of the middle-lower portion of the common bile duct and **(D)** dilatation of the upper biliary region on day 155 from baseline. ▸◂, duct diameter as measured by imaging.

After five months of treatment, the AFP and DCP decreased substantially to normal levels (**Figure 1B**), with undetectable levels of HBV DNA. On 19 July, contrast-enhanced magnetic resonance imaging demonstrated not merely tumor shrinkage but also the absence of intratumoral arterial enhancement (**Figure 1A**), which achieved a radiological complete response according to the modified RECIST (17). Moreover, magnetic resonance cholangiopancreatography revealed a marked stricture of the middle-lower portion of the common bile duct with dilatation of the upper biliary region compared with that observed during the pretreatment period (**Figures 3A–D**). Nevertheless, he had no clinical symptoms due to it, and thus, it was evaluated as grade 1 as per the Common Terminology Criteria for Adverse Events version 5.0 (18). As discussed by the MDT, he began withdrawing the anticancer medications and preparing for conversion surgery (14, 16).

**Figure 4:**
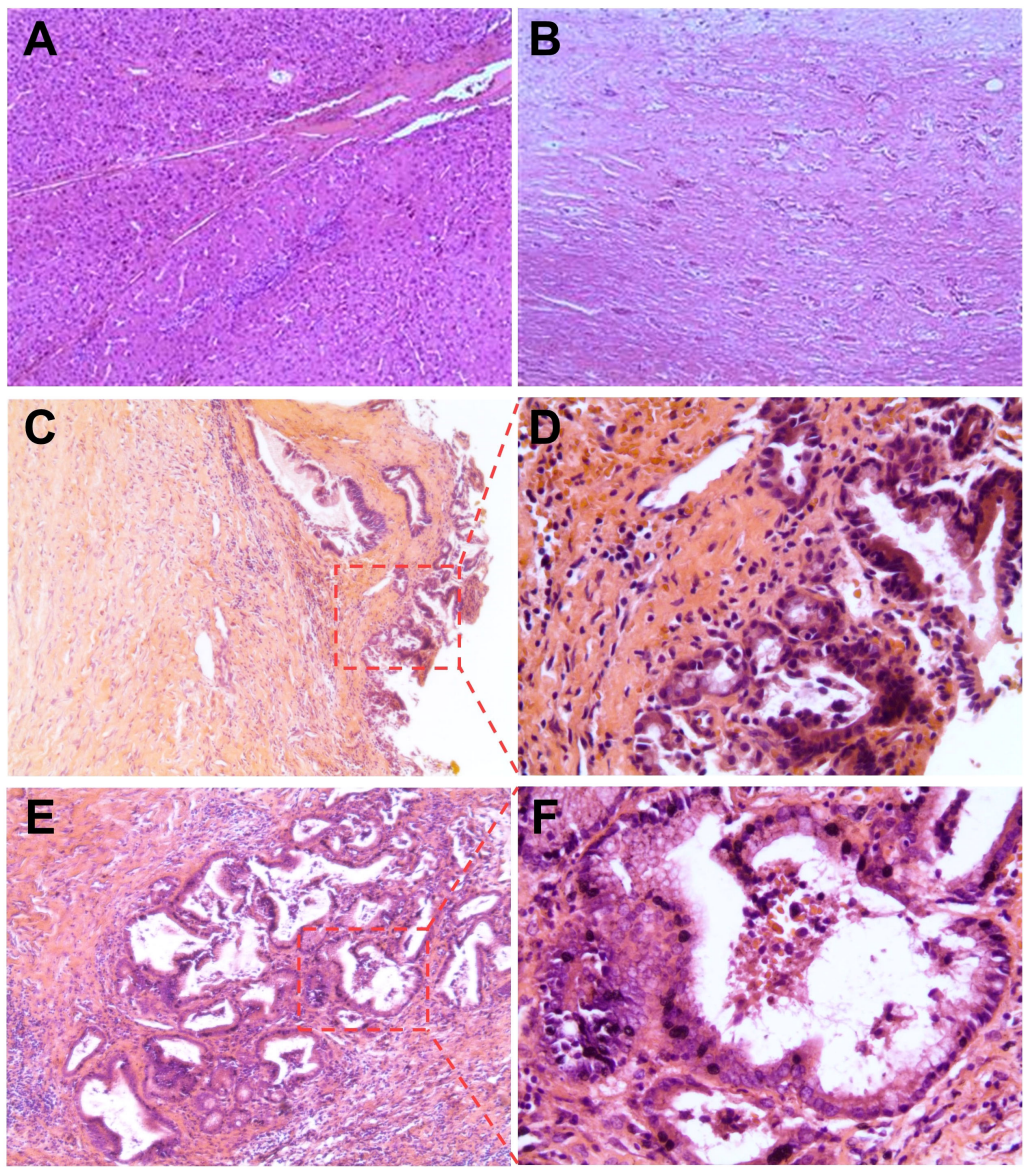
Hematoxylin and eosin staining of resected tumor and common bile duct biopsy tissues. **(A, B)** Histopathological examination of the resected tumor showing **(A)** large areas of necrosis in the center of the mass and **(B)** intrahepatic biliary hyperplasia and fibrosis in the surrounding hepatic tissues. **(C–F)** Common bile duct biopsy revealing **(C, D)** hyperplasia of the biliary epithelium and **(E, F)** severe inflammatory cell infiltration and fibrosis of the bile duct wall. Original magnification, 50× **(A–C, E)** or 200× **(D, F)**.

On 27 July, the patient underwent hepatectomy with microscopically negative surgical margins—an R0 resection. Furthermore, histopathological examination showed large areas of necrosis in the center of the resected mass with fibrosis and intrahepatic biliary hyperplasia in the surrounding hepatic tissues (**Figures 4A, B**). Of note, there was no residual microscopic tumor—a pathologic complete response. In addition, a biopsy of the middle-lower portion of the common bile duct revealed hyperplasia of the biliary epithelium, fibrosis of the bile duct wall, and severe inflammatory cell infiltration (**Figures 4C–F**).

After conversion surgery, the patient resumed the administration of lenvatinib and received another TACE procedure on September 11 as adjuvant therapy to prevent recurrence, whereas he no longer took tislelizumab given the risk of irAEs (16, 19). He is satisfied with the treatment and has regular follow-up visits in our outpatient department. According to follow-up examinations, his biliary stricture has resolved after discontinuation of tislelizumab, and no recurrence or other adverse events have been reported thus far.

## Discussion

In this report, we present a case in which massive unresectable HCC successfully achieved a complete response and R0 resection after quadruple conversion therapy directed by an MDT. Yet, despite considerable efficacy, this patient exhibited unexpected inflammatory cholangiopathy of the common bile duct and pseudoprogression in the AFP and DCP, which deserve special attention and are discussed below.

Drug-induced liver injury is common during the treatment of HCC, while ICI-induced large-duct cholangiopathy is infrequent (16, 20, 21). Without standard tests for irAEs, diagnosing irAEs remains challenging and largely relies upon the exclusion of alternative etiologies (19, 22). Chemotherapy-induced cholangiopathy occurs predominantly in the intrahepatic or perihilar biliary ducts but rarely in the common bile duct, and lipiodol deposition can be noted in the wall of bile ducts for TACE-induced cholangiopathy (23–25). By contrast, no lipiodol deposition was observed in our case, and the biliary stricture developed only in the middle-lower portion of the common bile duct (**Figures 3C, D**). Furthermore, anatomical distinction may explain the differential susceptibility of biliary regions to locoregional chemotherapy: upper biliary tree and HCC are primarily supplied by branches of the right/left hepatic arteries, which are selectively targeted during TACE/HAIC, whereas the middle-lower portion of the common bile duct indirectly receives blood from gastroduodenal artery (24–26). Regarding lenvatinib, hardly any similar adverse events were reported in the era when HCC treatment was dominated by TKIs (27, 28). In this study, our patient stopped the administration of tislelizumab because irAEs could not be ruled out (16, 19). Fortunately, his biliary stricture was observed radiologically only—asymptomatic—and resolved after withdrawal from the ICI. Taken together, biopsy, imaging, and resolution of stricture after ICI discontinuation supported the diagnosis of immune-related cholangitis (**Figures 3**, **4**). Moreover, similar observations were made in other types of cancer treated with ICI-based therapeutics, and it is intriguing that these studies suggest that liver enzymes might be predictive biomarkers of irAEs (20, 29, 30). In HCC, however, TACE and HAIC can cause fluctuations in the levels of liver enzymes (25); consequently, this utility of liver enzymes might be difficult to implement. Robust biomarkers to predict irAEs and guide clinical decisions are still needed (19, 22). In previous studies, the incidence of several adverse events—e.g., gastric ulcers—was considerably increased, albeit with better efficacy, in patients receiving quadruple conversion therapy than in those receiving TACE alone; nonetheless, no treatment-related adverse events similar to the present case were reported in these studies (10, 11). In clinical practice, the incidence of adverse hepatobiliary irAEs may be underestimated because of inadequate monitoring (9). Accordingly, adopting an MDT model can be important for patient-tailored therapeutics and the management of irAEs (5, 31, 32). Together, closer attention should be given to potential irAEs in patients receiving combination regimens involving ICIs, and more clinical trials are essential for broadening the basis for clinical decision-making and advancing this field.

On top of irAEs, pseudoprogression poses a growing challenge for patients and clinicians, and ICIs have profoundly revolutionized the management of HCC (6, 33). Pseudoprogression complicates tumor response assessment during ICI treatment, and there is an urgent need to establish reliable biomarkers to track clinical response (33, 34). Conventional radiological assessment criteria (i.e., RECIST) are insufficient to differentiate pseudoprogression from true progression, and the former usually occurs in the first few weeks after initiating ICI treatment and can lead to premature discontinuation of ICIs and underestimation of the efficacy of therapeutics (8, 34). Therefore, several new criteria have been proposed for ICI-based therapeutics, such as immune-related RECIST and immune-modified RECIST (34–36). Most of these criteria are based on imaging, while recent criteria, called RecistTM, are based on tumor markers (15). RecistTM could assess tumor response more efficiently than immune-related RECIST, as tumor markers might have a shorter response time and provide a more accurate prediction of overall survival compared to imaging (15). Interestingly, a total of 4 of the 70 lung cancer patients exhibited rapid increases followed by decreases in tumor marker levels within 3–6 weeks after ICI treatment (15), which is analogous to what our patient experienced (**Figure 2**). A similar pattern was observed in PIVKA-II (i.e., DCP) of another HCC patient treated with quadruple conversion therapy; nevertheless, this pattern has not received any attention (37). Notably, tumor marker pseudoprogression has also been observed in gastric cancer, in addition to lung cancer and HCC, suggesting it could be a broader phenomenon across cancer types (15, 37–39). Mechanistically, this phenomenon may be associated with inflammatory reactions, considering that benign inflammatory diseases—e.g., colitis and hepatitis—can also present with elevated AFP and DCP (40–42). The last several decades have seen a plethora of studies on the utility of AFP and DCP in the surveillance and management of HCC, whereas there are only limited studies on the predictive and monitoring role of these two biomarkers in ICI-based therapeutics (43). Based on 44 retrospective studies, a pooled analysis revealed that low baseline AFP and DCP levels and early AFP response might be associated with good outcomes in HCC patients treated with ICIs (44). More recently, a retrospective study reported that trends in AFP during the first three weeks after initiation of treatment and baseline DCP levels could be used to predict which patients would benefit from first-line ICI-based therapy (45); however, poor outcomes might be incorrectly predicted in cases with tumor marker pseudoprogression. With high baseline AFP and DCP levels, our patient achieved a complete response, although he presented tumor marker pseudoprogression during the three weeks after the first dose of tislelizumab (**Figure 1B**). On the other hand, substantially decreased AFP and DCP overall reflected the remarkable efficacy of the quadruple conversion therapy (**Figure 1B**), which suggests that serial AFP and DCP measurements are promising for monitoring the impact of therapeutic interventions for HCC. Given the lack of prospective evidence, the utility of AFP and DCP in monitoring and predicting clinical benefit from ICIs awaits validation in large-scale carefully designed studies.

In summary, this case demonstrated the great potential of quadruple conversion therapy for HCC, but presented unusual cholangitis and tumor marker pseudoprogression, both of which are associated with ICIs. Further evaluation, therefore, is warranted to assess the safety and generalizability of combination therapy and the feasibility and power of biomarker-guided treatments.

## Data Availability

The original contributions presented in the study are included in the article. Further inquiries can be directed to the corresponding authors.
